# Author Correction: Dephasingless laser wakefield acceleration in the bubble regime

**DOI:** 10.1038/s41598-024-55489-5

**Published:** 2024-02-28

**Authors:** Kyle G. Miller, Jacob R. Pierce, Manfred V. Ambat, Jessica L. Shaw, Kale Weichman, Warren B. Mori, Dustin H. Froula, John P. Palastro

**Affiliations:** 1 Laboratory for Laser Energetics, University of Rochester, Rochester, NY 14623-1299 USA; 2 Department of Physics and Astronomy, University of California, Los Angeles, CA 90095 USA; 3 Department of Electrical and Computer Engineering, University of California, Los Angeles, CA 90095 USA

Correction to: *Scientific Reports* 10.1038/s41598-023-48249-4, published online 02 December 2023

The original version of this Article contained errors in Figure 2c. Due to a misalignment of the data in panel (c), relative to the data in panel (b), the injection of argon electrons at the onset of the He/Ar region was visualized incorrectly.

The original Figure 2 and accompanying legend appear below.

**Figure 2:**
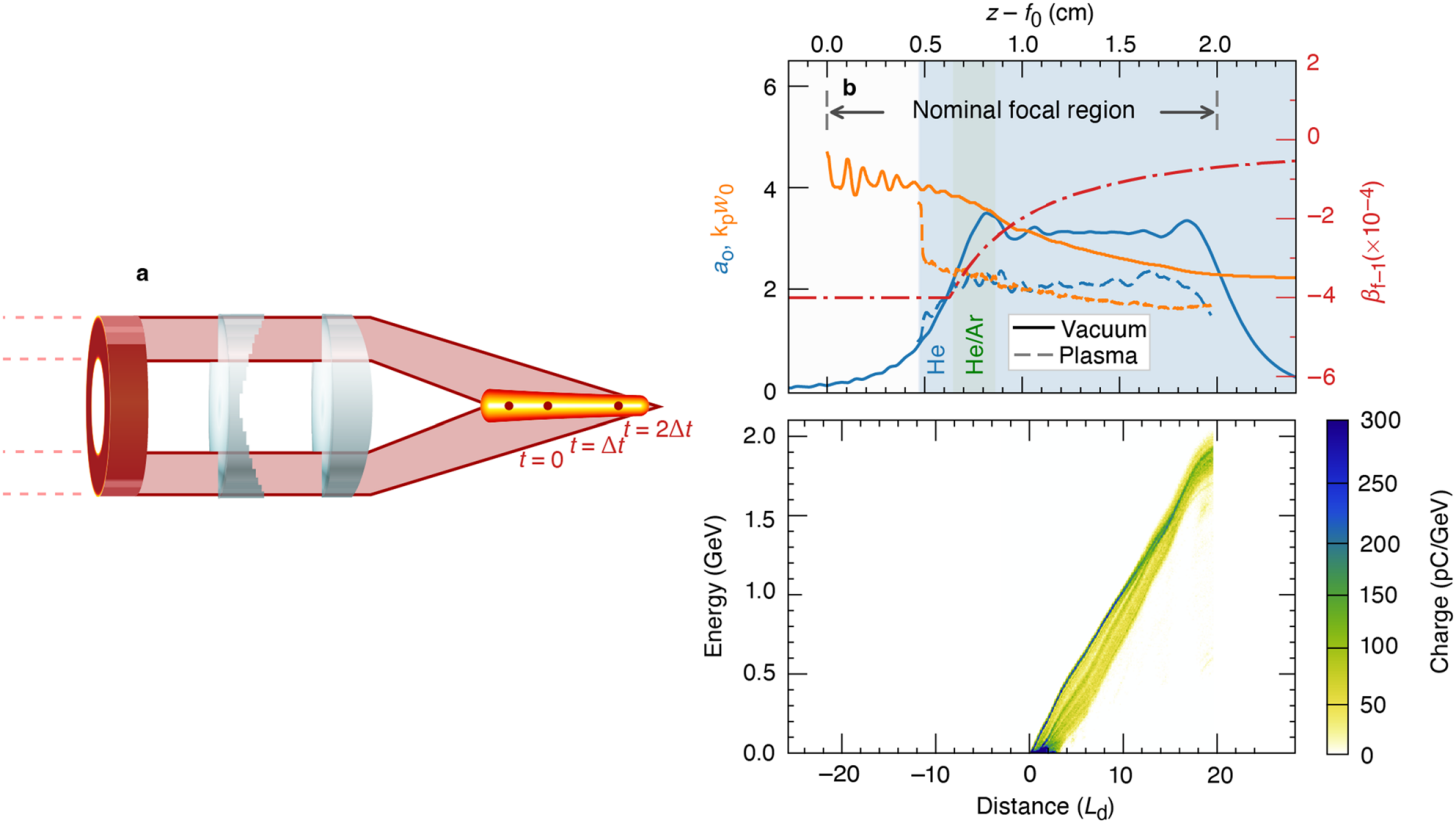
The ultrafast flying focus and electron acceleration in a bubble-regime dephasingless laser wakefield accelerator. (**a**) Schematic of the optical configuration for an accelerating focus, including the axiparabola and echelon. For illustrative purposes, the optics are shown in transmission, but experiments would likely be performed in reflection^30^. (**b**) The accelerator geometry showing the on-axis amplitude ${a}_{0}$  and inner-core spot size ${w}_{0}$ of the masked laser pulse—simulated in vacuum (solid) and plasma (dashed)—along with the designed focal velocity in the plasma ${\beta }_{{\text{f}}}$ (dot-dashed). (**c**) Energy gain of the ionization-injected electrons in the first bubble. After 20 dephasing lengths, 25 pC of charge was accelerated up to 2.1 GeV.

The original Article has been corrected.

